# Optical investigation and computational modelling of BaTiO_3_ for optoelectronic devices applications

**DOI:** 10.1038/s41598-023-31652-2

**Published:** 2023-03-23

**Authors:** Maryam G. Elmahgary, Abdelrahman M. Mahran, Moustafa Ganoub, Sameh O. Abdellatif

**Affiliations:** 1 The Chemical Engineering Department, British University in Egypt (BUE), Cairo, 11387 Egypt; 2 The Electrical Engineering Department, and FabLab, at the Centre of Emerging Learning Technologies CELT, British University in Egypt (BUE), Cairo, 11387 Egypt; 3 The Renewable Energy Postgraduate Programme, and FabLab, at the Centre of Emerging Learning Technologies CELT, British University in Egypt (BUE), Cairo, 11387 Egypt

**Keywords:** Electrical and electronic engineering, Chemical engineering

## Abstract

ABX_3_ perovskite-based materials have attracted research attention in various electronic and optoelectronic applications. The ability to tune the energy band gap through various dopants makes perovskites a potential candidate in many implementations. Among various perovskite materials, BaTiO_3_ has shown great applicability as a robust UV absorber with an energy band gap of around 3.2 eV. Herein, we provide a new sonochemical-assisted solid-phase method for preparing BaTiO_3_ thin films that optoelectronic devices can typically be used. BaTiO_3_ nano-powder and the thin film deposited on a glass substrate were characterized using physicochemical and optical techniques. In addition, the work demonstrated a computational attempt to optically model the BaTiO_3_ from the atomistic level using density functional theory to the thin film level using finite difference time domain Maxwell's equation solver. Seeking repeatability, the dispersion and the extinction behavior of the BaTiO_3_ thin film have been modeled using Lorentz-Dude (LD) coefficients, where all fitting parameters are listed. A numerical model has been experimentally verified using the experimental UV–Vis spectrometer measurements, recording an average root-mean-square error of 1.44%.

## Introduction

Relatively high band-gap semiconductors are significantly used in various optoelectronic devices^1–3^. Although the energy band-gap of a wide band-gap semiconductor may exceed the Shockley–Queisser limit, it can still be utilized as a front UV filter^4–7^. A UV filter can be functionalized in optoelectronic devices, specifically solar cells and light harvesters, to protect the solar cells from high-energy photons^8,9^. For example, the new generation of solar cells, including but not limited to perovskite solar cells^10^, dye-sensitized solar cells^11^, and organic solar cells^12^, have shown severe degradation under UV emissions^8^. Accordingly, UV absorbers are commonly used as a protective layer^6,13^. Broad band-gap UV filters can be integrated into multifunction tandem cells as a front layer^14,15^.

BaTiO_3_ is considered one of the low-preparation cost alternatives for wide band-gap semiconductors^16–21^. Typically, the energy band gap of BaTiO_3_ is varied from 3.2 to 3.4 eV^22–25^. Attempts were introduced in the literature to modulate the band gap, seeking visible absorption^23,24^. However, the wide band-gap BaTiO_3_ is still very beneficial as a UV absorber^26,27^, especially in perovskite solar cells^28,29^, as reported in^20^. Another work utilized BaTiO_3_ in perovskite thin films but with Si-doping^25^ and other dopants^30^. Moreover, BaTiO_3_ recorded efficient integration in dye-sensitized solar cells, following data presented in^16,18,31,32^. Over and above, the UV absorption capability can be considered a credit in designing indoor light harvesters, where the UV portion in the light spectrum dominates^33,34^. In addition to the interesting optical properties of BaTiO_3_, it also showed exciting features in other applications, such as gas sensing^17^, water treatment^35^, and piezo-photoelectronic coupling^18^.

Recently, the optical and electrical properties of barium titanate (BaTiO_3_) have attracted a wide range of researchers, as demonstrated in the literature^36–42^. The impact of the synthesizing process on the optical properties of the BaTiO_3_ nano-structure is studied in^37^. Consequently, the optical band gap and the refractive index of the nanoparticles are well investigated in work reported in^36,39^. The influence of the compressive strain on the optical properties of BaTiO_3_ is tackled in^43^. The recorded data showed a variation in the refractive index from 1.55 to 1.65^36^. Sb–BaTiO_3_ and Y–BaTiO_3_ doped ceramics prepared by solid-state reaction were optically investigated in^44^. Another doped version of BaTiO_3_, Ce-Doped BaTiO_3_, was discussed optically and electronically in^45^ using first-principles calculations. Applying the same approach, the work in^46^ studied the Cr-doped BaTiO_3_. Alternatively, the utilization of density function theory (DFT) simulations glows up to accurately calculate the energy band-gap of the material^47^. Using hybrid HSE06 functional, the calculated band-gap values were 3.254, 3.894, 3.694, 3.519, and 3.388 eV, corresponding to the cubic, rhombohedral, and orthorhombic, respectively tetragonal, and hexagonal phase of BaTiO_3_ polymorphs^47^.

In the current investigation, BaTiO_3_ nano-powder has been prepared using a novel sonochemical-assisted solid-phase method and deposited over a BK7 glass substrate. The sonochemical-assisted solid-phase method overcomes the shortcomings of too high temperature in solid-phase separation. The adopted method is a simple, direct, and ultrasonic treatment to ensure the homogeneity of the suspension. For material characterization, the samples were characterized using X-ray diffraction (XRD), Fourier-transform infrared spectroscopy (FTIR), and atomic force microscopy (AFM). Optically, the diffuse reflectance spectroscopy (DRS) measurement was conducted for band-gap calculation, and UV–Vis-NIR spectrometer measurements were utilized to capture the optical transmission. The finite difference time domain solver was used to model the UV–Vis spectrum, whereas the density function theory model generated the optical permittivity as a complex wavelength-dependent function.

## Experimental work

**Figure 1:**
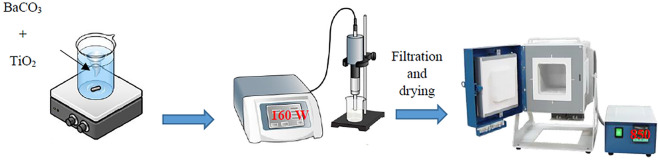
Barium titanate powder preparation setup.

A novel sonochemical-assisted solid-phase method for the preparation of nano BaTiO_3_ is proposed in this manuscript to solve the shortcomings of ultra-high temperature in solid-phase preparation (see Fig. 1). BaCO_3_ was dispersed in water with TiO_2_ (molar ratio: TiO_2_:BaTiO_3_ = 1:1, as reported in^48^). Stirring was done at 35 °C for 30 min. More details about the TiO_2_ recipe can be accessed in our previous work^49–53^. After that, ultrasonic treatment was performed for 30 min, and the power was 160 W. Then, the formed powder was filtered and dried by heating at 80 °C for 8 h. After grinding, the BaTiO_3_ was prepared by sintering at 850 °C for 5 h, during which the heating rate was 5 °C per minute. BaTiO_3_ was formed according to the following chemical reactions by Beauger et al.^54^.

1 $${\text{BaCO}}_{{3}} \to {\text{ BaO }} + {\text{ CO}}_{{2}}$$

2 $${\text{2BaO}} + {\text{ TiO}}_{{2}} \to {\text{ Ba}}_{{2}} {\text{TiO}}_{{4}}$$

3 $${\text{Ba}}_{{2}} {\text{TiO}}_{{4}} + {\text{TiO}}_{{2}} \to {\text{ 2BaTiO}}_{{3}}$$

**Figure 2:**
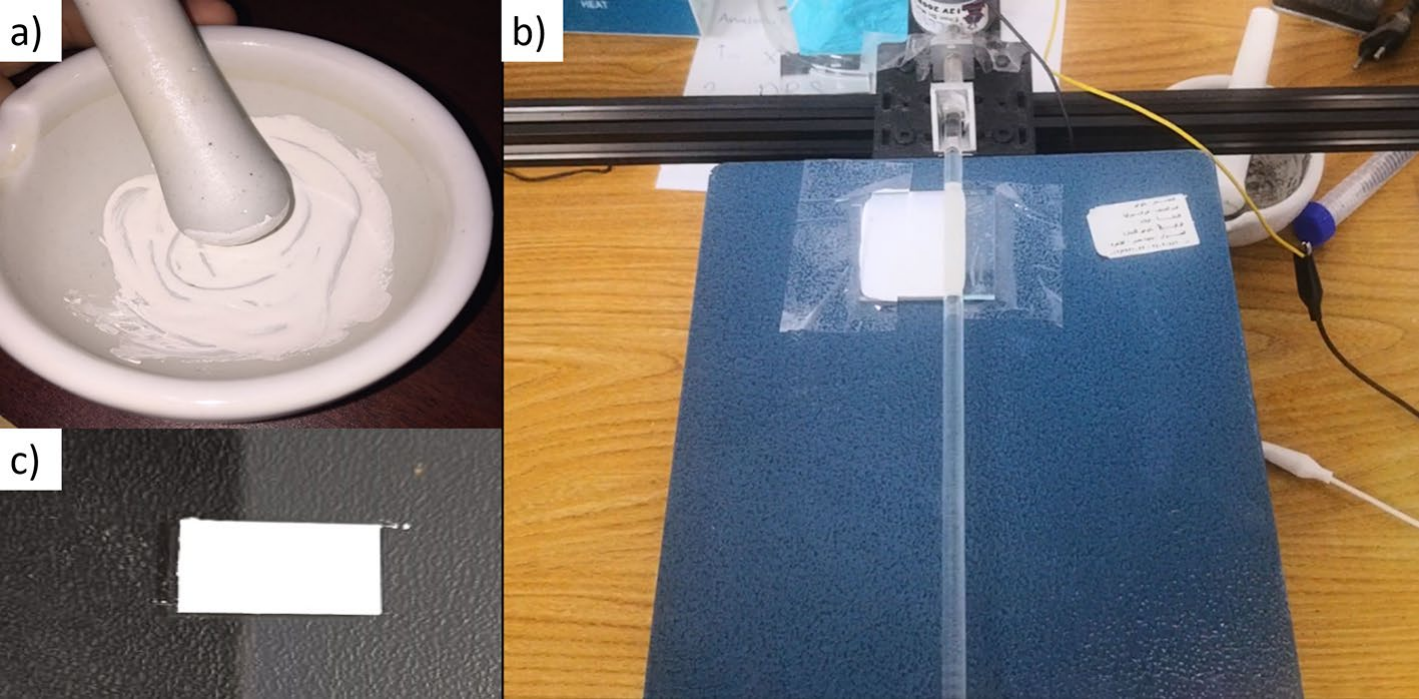
(**a**) Barium titanate paste, (**b**) automatic screen printing deposition, and (**c**) BaTiO_3_ thin film on a glass substrate.

For thin film deposition, about 0.5 gm of the TiO_2_ white powder in the mortar is mixed with 2–3 drops of the viscous colorless liquid carboxy methyl cellulose sodium salt (CMC) polymer slowly. The function of CMC is that it binds the BaTiO_3_ powder to the surface of the glass. Viscous CMC is prepared by dissolving 1 g of CMC powder in 250 mL distilled water in a beaker and placing this beaker on the magnetic stirrer for 4 h at 50 °C and 100 rpm. Grind and mix them with the pestle until a white paste is formed. On a non-conductive glass (5 cm × 1 cm), the substrate is fixed using a thin tape in order to have the thickness of the BaTiO_3_ paste layer at the same thickness. The used substrate is cleaned for 30 min with the water Labosol solution and then with distilled water, Followed by another 30 min in a Water–ethanol solution of NaOH, then distilled water again. Finally, dry the samples using the N_2_ stream. The screen printing is done by adding a few drops of BaTiO_3_ paste to the glass and spreading it evenly to the thickness of the tape using an automated glass rod screen printing machine, previously customized and reported in^51^. Finally, the glass substrate is placed on a hot plate for 6–8 min at 130 °C, removed, and left to cool down at room temperature. The complete process is illustrated in Fig. 2. Herein; repeatability is ensured as the printing process is managed automatically.

Thin film deposition for the prepared BaTiO_3_ is conducted using our customized screen-printing tool^51^. In such a setup, the layer thickness can be roughly controlled through the motor biasing voltage, which reflects on the glass rod speed. The material charismatics of the prepared samples were measured using XRD (Empyrean Malver Panalytical), AFM (alpha300 Atomic Force Microscope from WITec GmbH), and Fourier Transform Infrared (FTIR) spectroscopy Burker Vertex70. Optically, the optical transmission spectra of the fabricated samples are measured through a V-770 UV–Vis.-NIR Spectrophotometer, Cary 5000, with a wavelength range from 190 to 2700 nm, and Diffuse reflection spectra (DRS) were used for band-gap calculations.

### Consent to participate

All authors confirm their participation in this paper.

## Computational model

Herein, we utilize the density function theory (DFT) to estimate the energy band gap for the BaTiO_3_, which can be experimentally verified using the DRS measurements. Utilizing the same VASP procedure as in^50^, four input files, INCAR, POTCAR, POSCAR, and K-POINTS, were inserted into the model. As outputs, the HOMO–LUMO gap was presented to estimate the material energy band gap. Moreover, the material permittivity as a complex wavelength-dependent function was obtained against wavelength using the DFT post-calculation, given by:

4 $$\upvarepsilon \left(\uplambda \right) =\upvarepsilon ^{\prime } \left(\uplambda \right) -\upvarepsilon ^{\prime \prime } \left(\uplambda \right)$$

where ε(λ) is the material permittivity as a function of wavelength λ, ε′(λ) is the real part of the material permittivity, used in calculating the real material refractive index $n$, and ε″(λ) is the imaginary part of the material permittivity, exploring the optical extinction of the material.

**Figure 3:**
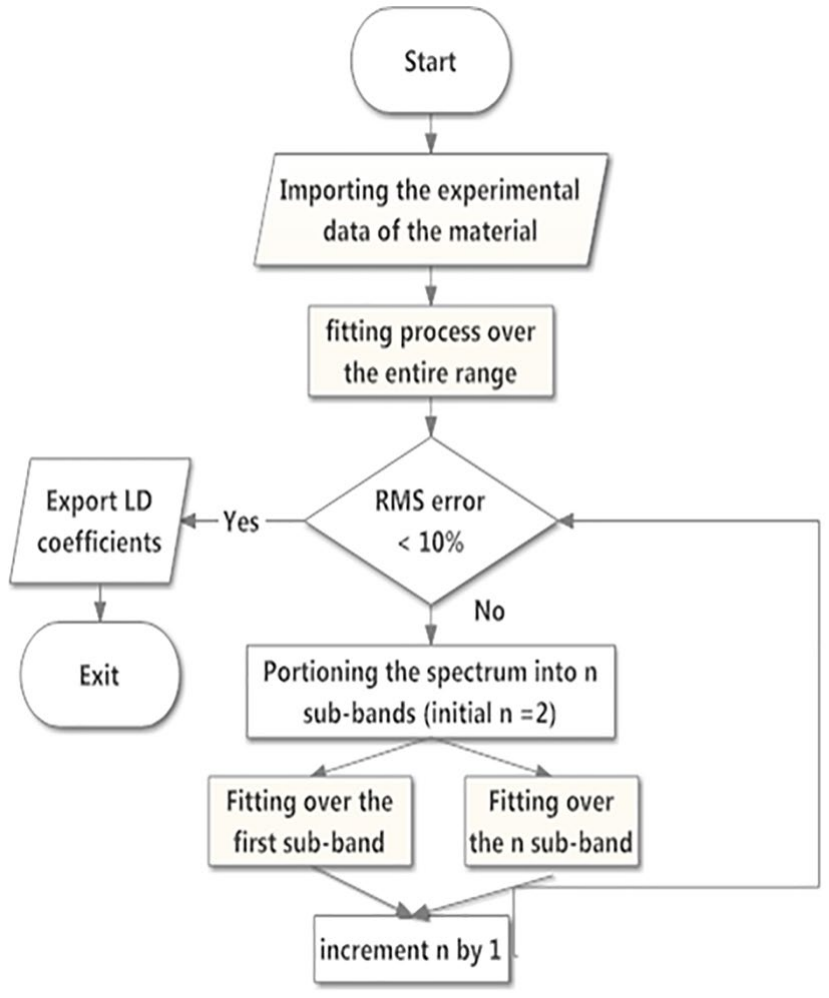
LD fitting algorithm using FDTD MEEP simulator.

Moving toward the thin film layer, an open-source, Linux-based Maxwell's equation solver, MEEP, was used to model the optical transmission spectrum using the finite difference time domain (FDTD) computational technique^55^. MEEP utilizes a scaled Maxwell's equations with a scaling factor of $a$. For an input in the form of a gaussian beam of peak wavelength at 550 nm, the field at the end of the layer can be derived from a simple matrix operation given by:

5 $$\left[ {\begin{array}{*{20}c} {E_{1} } \\ {H_{1} } \\ \end{array} } \right] = M{ }\left[ {\begin{array}{*{20}c} {E_{2} } \\ {H_{2} } \\ \end{array} } \right]{ }$$

where $E_{1}$ and $H_{1}$ are the electric and magnetic field intensity in the input medium, $E_{2}$, and $H_{2}$ are the electric and magnetic field intensity in the output medium, and *M* is given by:

6 $$M = { }\left[ {\begin{array}{*{20}c} {\cos k_{o} h} & {i\sin k_{o} h/\gamma_{1} { }} \\ {\gamma_{1} { }i\sin k_{o} h} & {\cos k_{o} h} \\ \end{array} } \right]{ }$$

and ${ }\gamma_{1}$ is given by:

7 $$\gamma_{{1{ }}} = { }\sqrt {\frac{{\varepsilon_{o} }}{{\mu_{o} }}} \frac{{n_{1} }}{{\cos \theta_{i} }}$$

where $k_{o}$ is the propagation coefficient ($k_{o} = {2}\uppi /\uplambda$), λ is the wavelength, h is the thin film thickness, ${\varepsilon }_{o}$ and ${\mu }_{o}$ are the air permittivity and permeability, ${n}_{1}$ is the refractive index of the thin film material and ${\theta }_{i}$ is the angle of incidence. The material dispersion and extinction spectra are inserted into MEPP using Lorentz–Drude (LD) coefficients^55^. These LD coefficients are the main link between the DFT and MEEP models. LD coefficients are extracted using the algorithm demonstrated in Fig. 3.

## Experimental results

This section demonstrates the experimental and numerical results for the material and optical properties of BaTiO_3_ thin film. Numerical investigations are integrated into this manuscript to provide a repeatable simulation parameter for BaTiO_3_ that can be useful for the research community in modeling BaTiO_3_ in various optical and optoelectronic devices. In addition, the proposed simulation model is verified in terms of band-gap for the DFT model and transmission spectra for the FDTD optical model concerning experimental measurements.

### Material characterization

**Figure 4:**
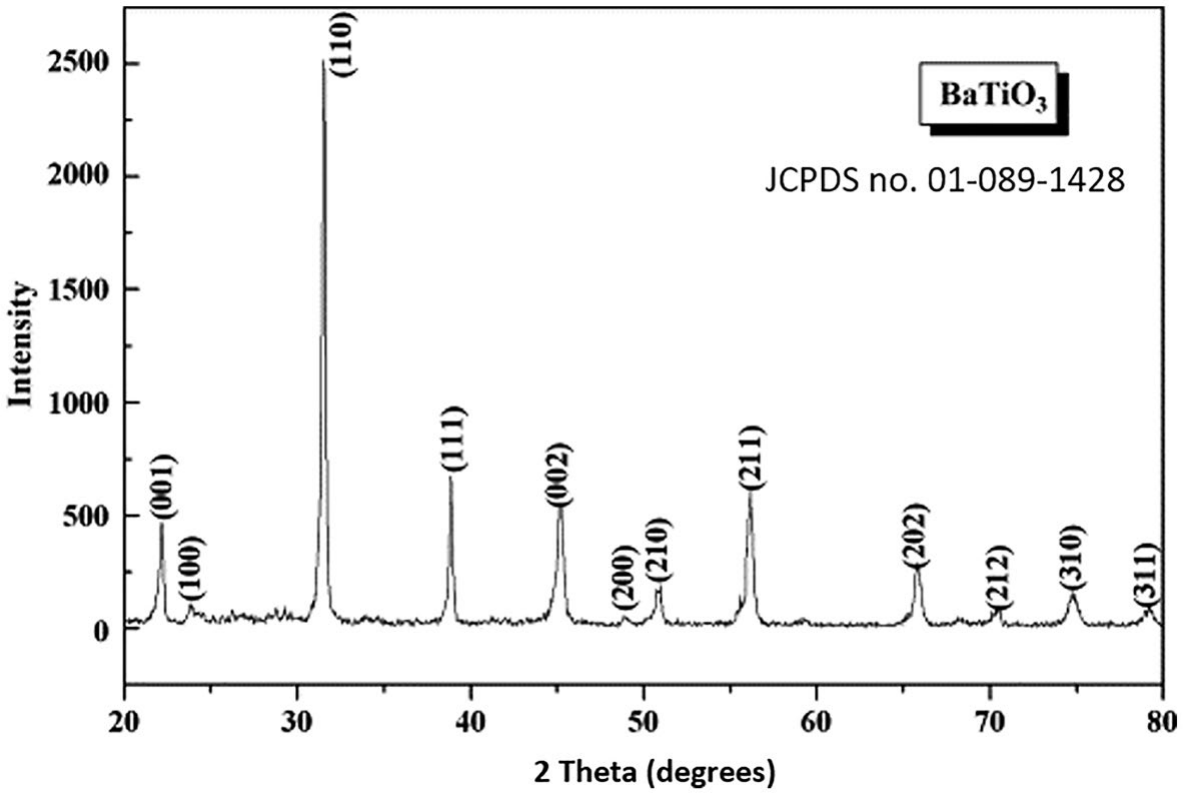
X-ray diffraction pattern for barium titanate powder JCPDS No. 01-089-1428).

Initially, the BaTiO_3_ nano-powder was investigated using XRD. XRD is a powerful technique commonly utilized to characterize nanomaterials to investigate crystalline properties, including the crystalline phases, the corresponding planes, the average crystalline sizes, and many other parameters. XRD pattern is represented in Fig. 4. The peaks at 22.14°, 31.5°, 38.8°, 45°, 50.8°, 56.1°, and 66.1° were ascribed to (001), (110), (111), (002), (210), (211) and (202) plane respectively which corresponding to BaTiO_3_ (JCPDS No. 01-089-1428). The size of BaTiO_3_ crystallites recorded is estimated by the Debye–Scherrer^56,57^:

8 $${\text{t}} = \frac{{{\text{K}}\uplambda }}{{\upbeta \cos\uptheta }}$$

where ${\text{K}}$ is the Scherrer constant of 0.89, ${\uplambda }$ denotes the wavelength of the X-ray source, ${\beta }$ denotes the full width at half maxima (FWHM), and ${\uptheta }$ denotes the Bragg’s diffracted angle. The crystallite size was recorded at 59 nm, with the aid of the top five peaks in the XRD illustrated in Fig. 4. Herein, we utilize the first five peaks as these sharp and resolved peaks showed the best-fitting statistics obtained. The lattice parameters $a$ and $c$ are calculated using the equation^56,57^:

9 $$\frac{1}{{d^{2} \left( {hkl} \right)}} = \frac{4}{3} \left[ {\frac{{h^{2} + hk + l^{2} }}{{a^{2} }}} \right] + \frac{{l^{2} }}{{c^{2} }}$$

This material crystallizes in a Tetragonal structure with lattice parameters a = 4.00 Å and c = 4.01 Å. The average lattice strain ε is determined by^56,57^:

10 $$\upvarepsilon = { }\frac{\beta }{4}\cos\uptheta$$

The average lattice strain showed ε = 0.202. The dislocation density δ is formulated by the equation^58–60^:

11 $$\updelta = \frac{{15\upbeta \cos\uptheta}}{4aD}$$

Consequently, the dislocation density was 1.76 × 10^9^ cm^-2^. The determined dislocation density agrees with previously reported data in the literature^61–63^. The work in^62^ has reported a variation in the dislocation density of the BaTiO_3_ from 1.7 × 10^9^ to 1.0 × 10^9^ cm^−2^, while the data produced in^63^ recorded the same order of magnitude of 10^9^ cm^−2^.

**Figure 5:**
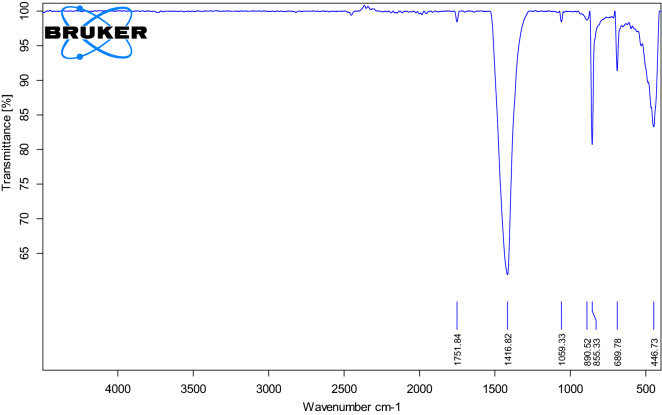
$FTIR$ of BaTiO_3_ powder, where the x-axis represents the wavenumber $k$ in cm^−1^, and the y-axis indicates the transmittance in %.

**Figure 6:**
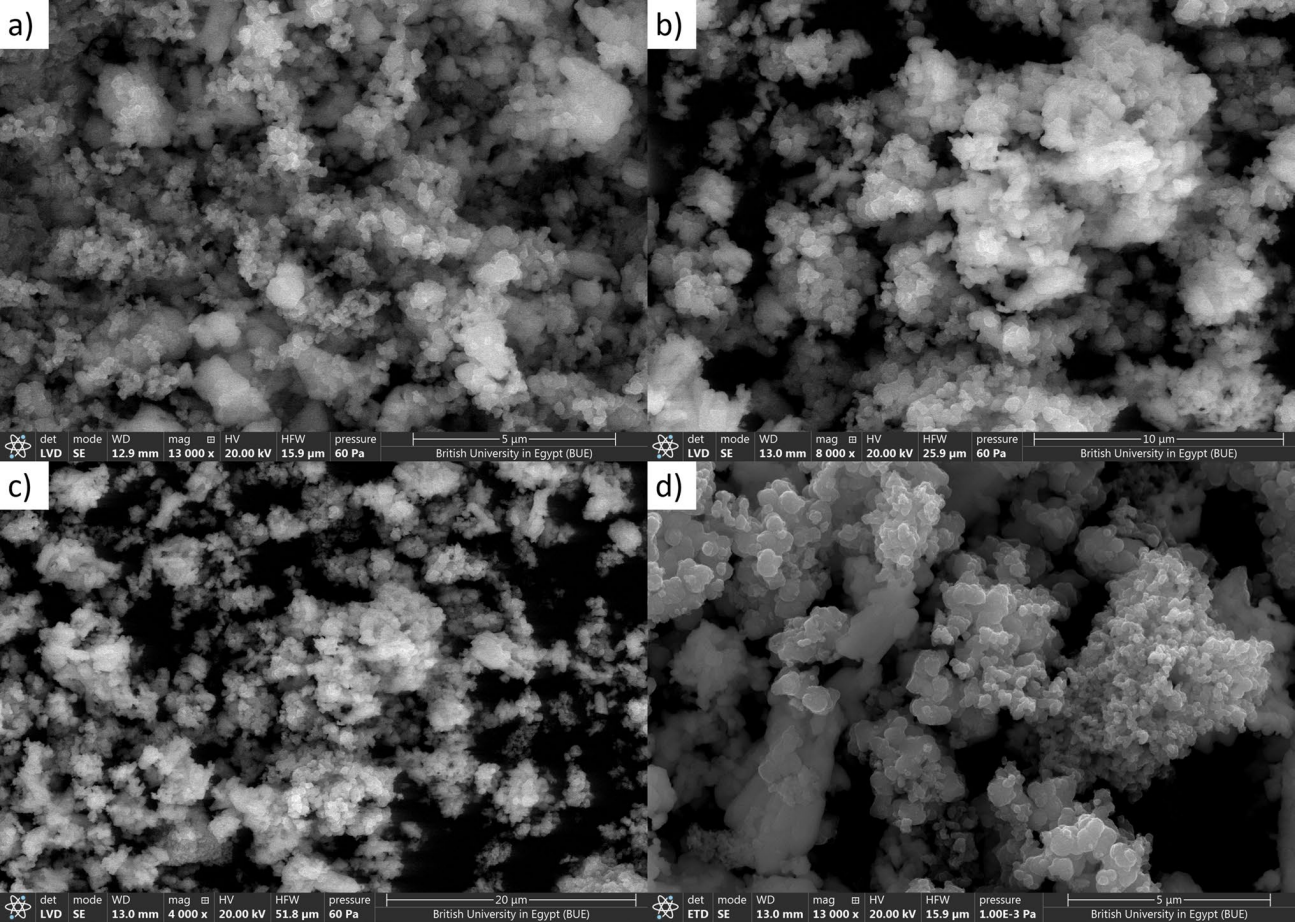
SEM measurements for BaTiO_3_ powder (**a**)–(**d**).

**Figure 7:**
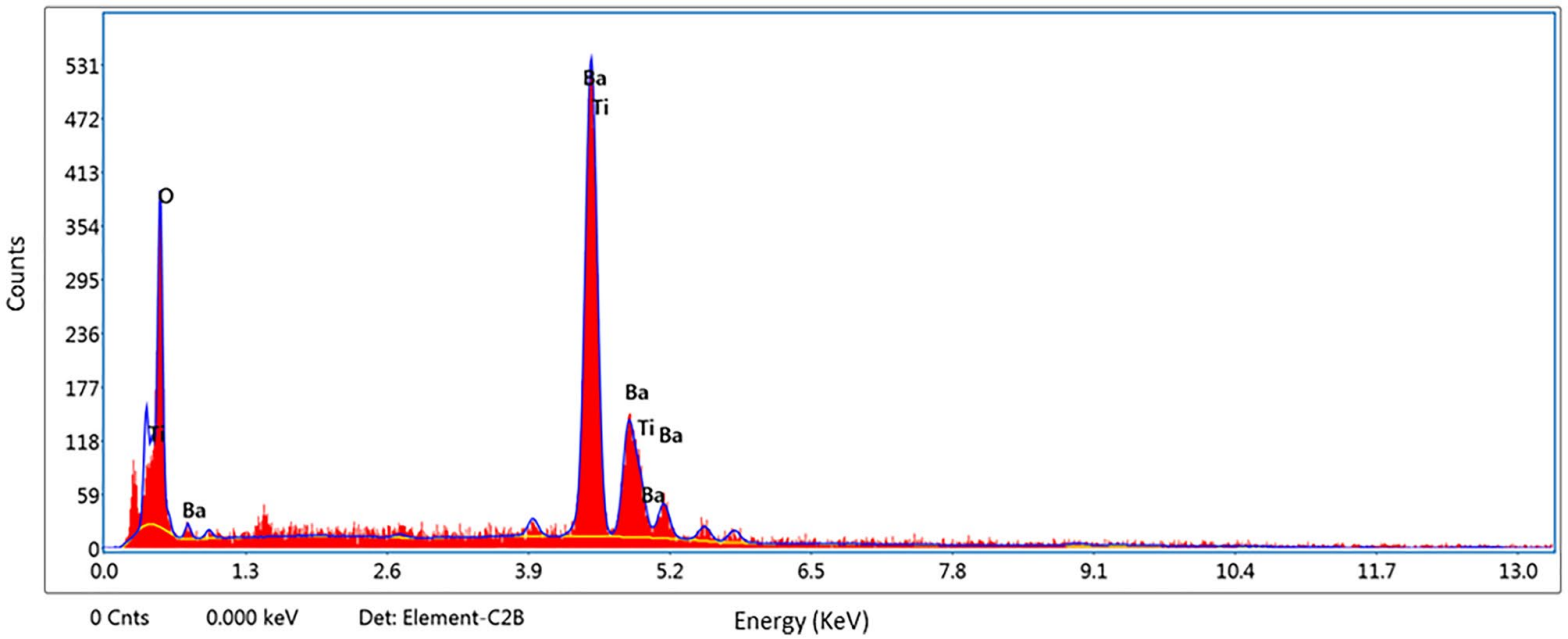
Energy dispersive X-ray analysis (EDX) measurements for BaTiO_3_ powder. Herein the x-variation is for the energy in KeV. At the same time, the y-variation indicates the number of counts, which reflects the material weight in the composition, as highlighted in Table 1.

**Figure 8:**
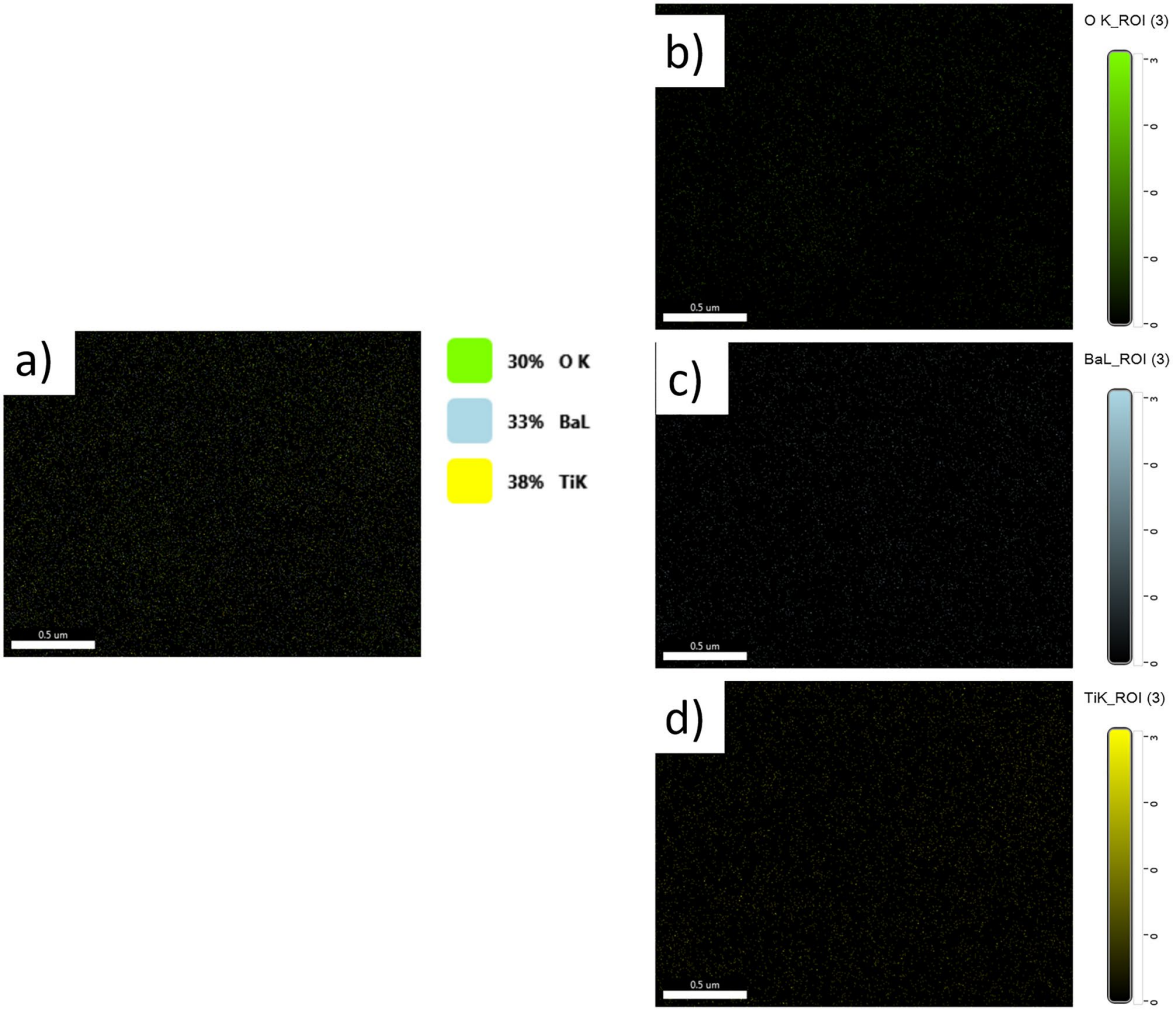
Mapping measurements for BaTiO_3_ powder.

**Figure 9:**
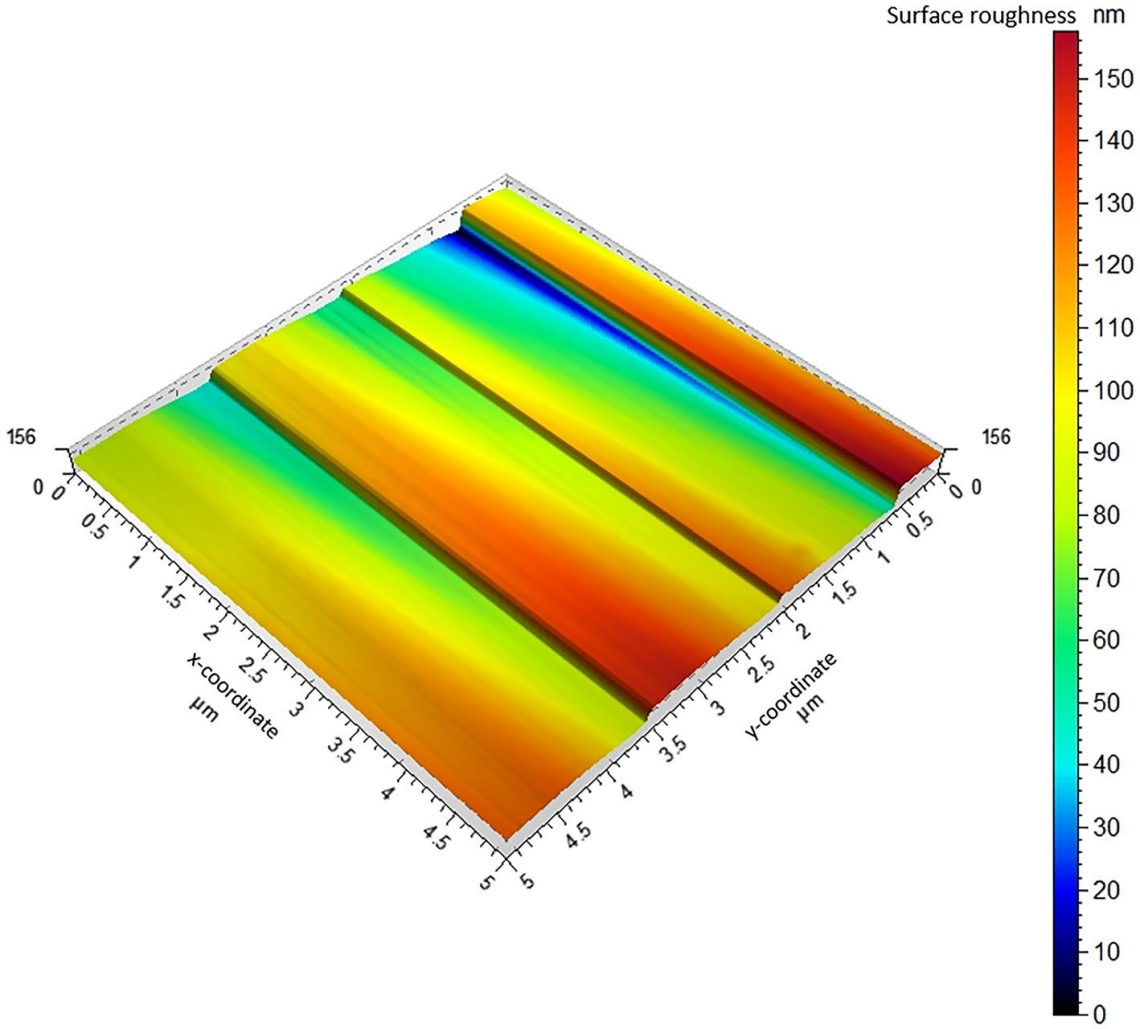
Roughness measurement of Barium Titanate Thin Film using atomic force microscope. The x–y axis represents the thin film coordinates in μm, while the color bar indicates the morphological surface roughness in nm.

The X-ray diffraction pattern shows that the synthesized product is single-phase, well-crystallized, and tetragonal BaTiO_3_. Additionally, Fig. 5 shows the FTIR spectra of nano-structure BaTiO_3_. The low-frequency region of the spectrum at 988 cm^−1^ is attributed to O–H bonded to titanium. The same trend of the 988 cm^−1^ bands was obtained in the strong absorption peak of asymmetric stretching carbonates ion (BaCO_3_) at 1434 cm^−1^. Additionally, the powder SEM characterization was carried out in Fig. 6. The SEM results showed that the grains of BaTiO_3_ is irregularly polygonal in shape. EDX analysis of particles calcinated at 850 °C is given in Fig. 7 and confirms the accuracy of elemental composition. The particles are composed of Ba, Ti, and O elements. The experimental values of the BaTiO_3_ sample obtained from the spectrum of energy dispersive X-ray analysis (EDX) are shown in Table 1, and the corresponding element mapping is in Fig. 8. Finally, the thin film roughness was measured using an atomic force microscope (AFM), cf. Fig. 9. The experimental data recorded an acceptable roughness, with an average root-mean-square variation of around 1.50 nm in a (0.5 μm)^2^ region. Thin film is deposited on a BK7 glass substrate using screen printing, as introduced in “Experimental work” section. The recorded surface roughness showed significant improvement to the data in the literature as in^64^. We attribute this to the sonochemical-assisted solid-phase method to prepare nano BaTiO_3_. Additionally, the deposited samples were monitored several weeks after deposition, where no color changes were detected.

**Table 1:** Experimental values for the Energy dispersive X-ray analysis (EDX) measurements for BaTiO_3_ powder.

Element	Weight (5%)	Atomic (%)	Error (%)
0 K	20.79	59.93	11.06
Ba L	57.72	19.38	6.15
Ti K	21.49	20.69	4.71

According to the table, the designation ‘K’ or ‘L’ is coupled to the excitation of the K or the L shell, which arises due to the recombination of the K- and L-shell vacancies.

**Figure 10:**
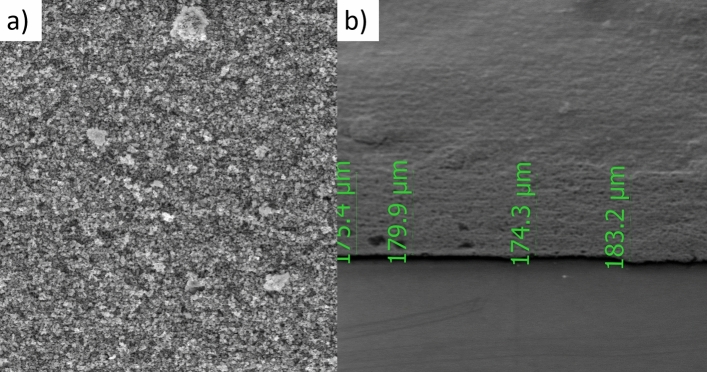
Thickness measurement of barium titanate thin film using SEM.

For the sake of thickness investigation, the SEM measurements in Fig. 10 (a demonstrated a top surface view, while b shows an edge view for thickness estimation) were conducted, showing an average film thickness of 178.45 μm, with the aid of the post-image processing technique and the correction factors introduced in our previous work in^65^. SEM measurement in Fig. 10a was also utilized to explore the porosity of the samples using a MATLAB image processing toolbox, as previously utilized in our work^66^. Herein, the processing showed a porosity of around 44.32%. Moreover, the SEM image in Fig. 10a is used to capture an estimated grain size of the BaTiO_3_ particles, where an adequate size of 23 nm is recognized. The image post-processing calculated grain size properly matches the one determined using the XRD in Fig. 4. We can attribute the slight mismatching to the aggregation effects associated with the solvent. All the obtained parameters in this section are listed in Table 2, where a comparison concerning literature is shown whenever possible.

**Table 2:** Experimentally obtained results for BaTiO_3_.

Parameter	Obtained via	Value	Comparison with literature
The crystallites size	XRD	59 nm	From 50 to 100 nm, as reported in^67^
The average lattice strain (ε)	XRD	0.202	Agrees with the data in^68^
The dislocation density	XRD	1.76 × 10^9^ cm^−2^	From 1.7 × 10^9^ to 1.0 × 10^9^ cm^−2^ in^62^, order of 10^9^ cm^-2^ in^63^
Thin-film roughness	AFM	1.50 nm in 0.5 μm^2^ region	10–20 nm, as reported in^64^
The average film thickness	SEM	178.45 μm	NA

### Optical characterization

**Figure 11:**
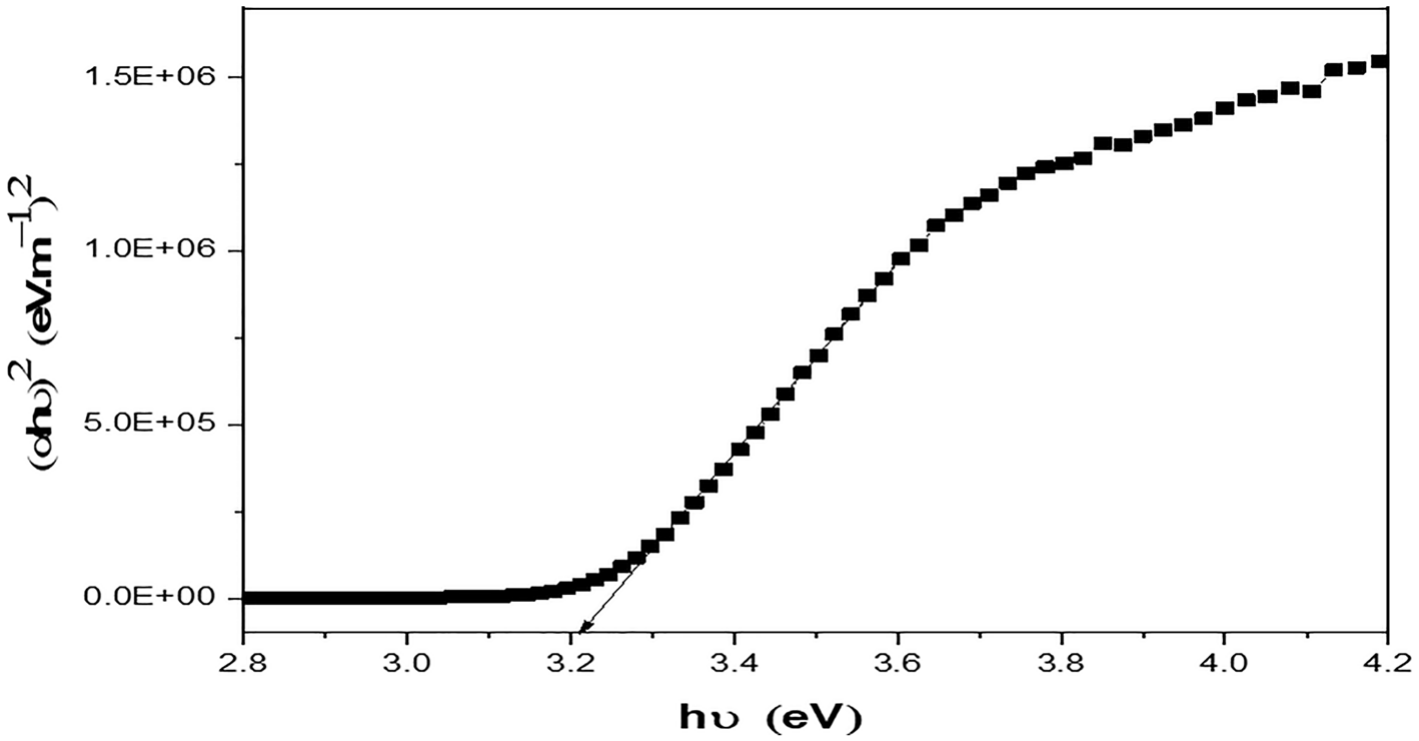
Tauc plot for barium titanate.

**Figure 12:**
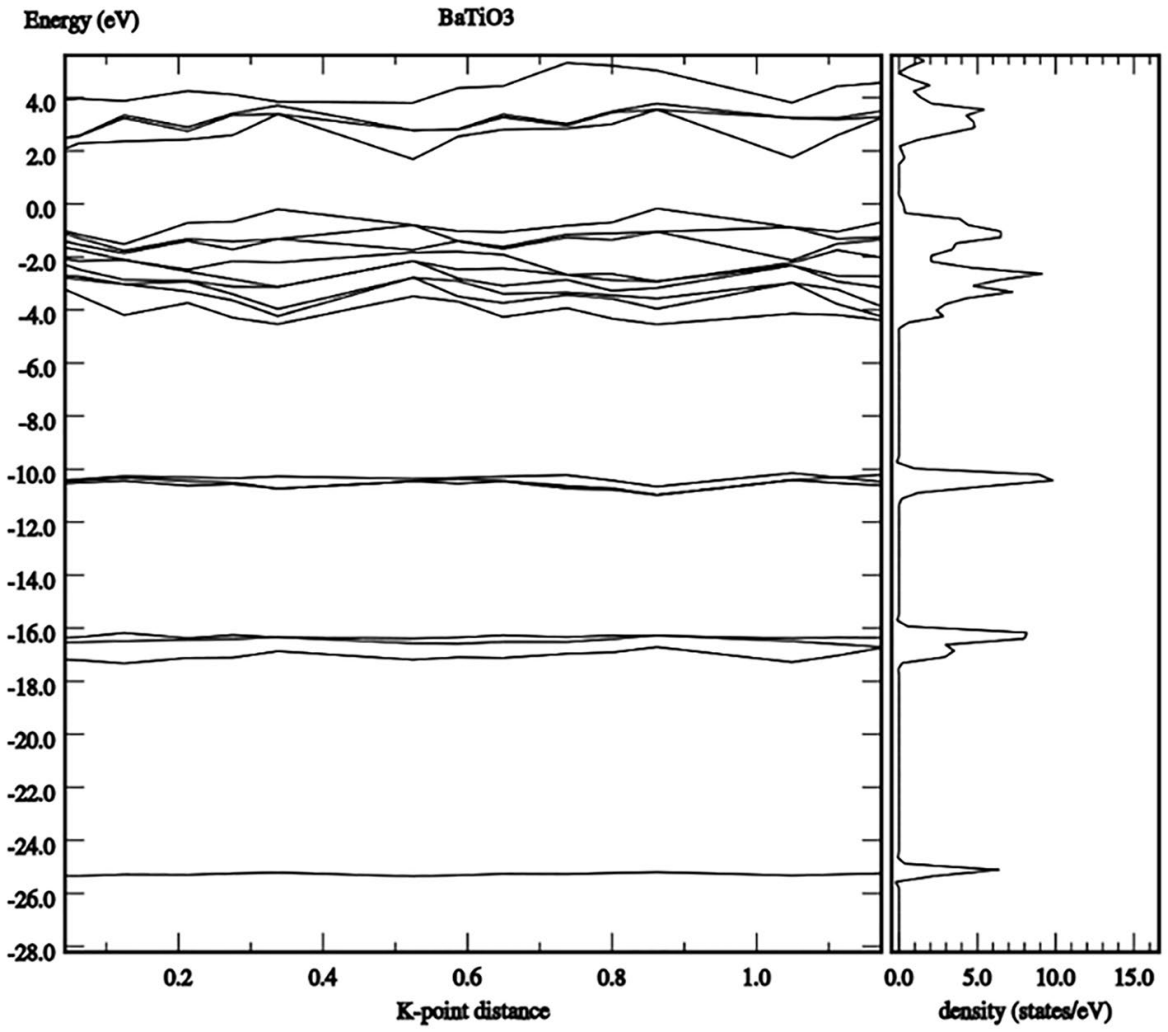
DOS and band structure for BaTiO_3_ outputted from the DFT VASP simulation model.

Principally, the diffuse reflection spectra (DRS) were measured to investigate the light absorbance profile of the prepared nanomaterial. Figure 11 shows the Tauc plot, where the Tauc relation is given by^56,57^:

12 $$\varepsilon^{^{\prime}} \left( {h\nu } \right) = C\left( {h\nu - E_{g} } \right)n$$

where C is a constant, $\varepsilon^{^{\prime}} \left( {h\nu } \right)$ is the molar extinction coefficient, $E_{g}$ is the average band gap of the material, and n depends on the type of transition. The band gap of prepared BaTiO_3_ nanoparticles was estimated to be 3.2 eV. Alternatively, DFT modeling is conducted, as mentioned in “Consent to participate” section. Perovskites are famous for the ABX_3_ structure, where a three-dimensional corner-sharing BX_6_ octahedron is formed. For simplicity, we consider only the cubic structure to explore the optoelectronic properties of BaTiO_3_. Firstly, the ABX_3_ structure is relaxed to calculate the lattice constant. Accordingly, the flexible structure is simulated to demonstrate the energy band diagram and the density of states (DOS) shown in Fig. 12. Knowing that the generalized gradient approximation (GGA) underestimates the band-gaps in DFT simulations, the hybrid nonlocal exchange–correlation functional (HSE) is used to calculate the band-gap of the BaTiO_3_ accurately. By observing the DOS, it can be concluded that BaTiO_3_ is an indirect band-gap material with a typical band-gap of 3.212 eV. This validates our simulation process, as it matches our experimental DRS measurements in Fig. 9 and reported data in the literature^25,35,69,70^.

**Figure 13:**
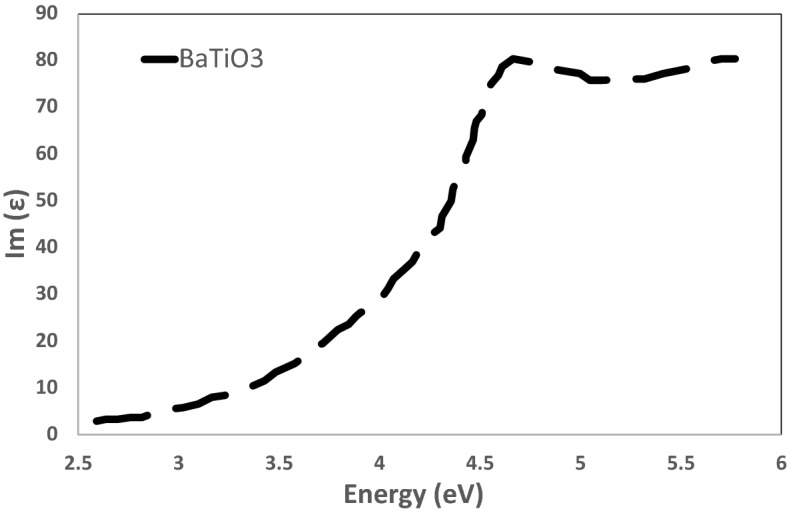
The imaginary permittivity component $(\varepsilon^{\prime \prime } ){ }$ for BaTiO_3_, as outputted from the DFT VASP simulation mode.

Next, the imaginary part of the relative permittivity is simulated for BaTiO_3_ in Fig. 13. This spectrum reflects the extinction behavior of the materials in the optical region of interest from 200 to 550 nm. Herein, we can assume a dominating absorption effect over other scattering mechanisms. This can be easily proven due to the limited surface roughness measured by the AFM in Fig. 9. Thus, the surface scattering can be neglected. The real part of the permittivity is also extracted for refractive index estimation. The refractive index showed 1.595, which agrees with the reported data in^36^. Consequently, the data simulated in Fig. 13 can be directly converted to LD coefficients to be fed into the MEEP model.

**Figure 14:**
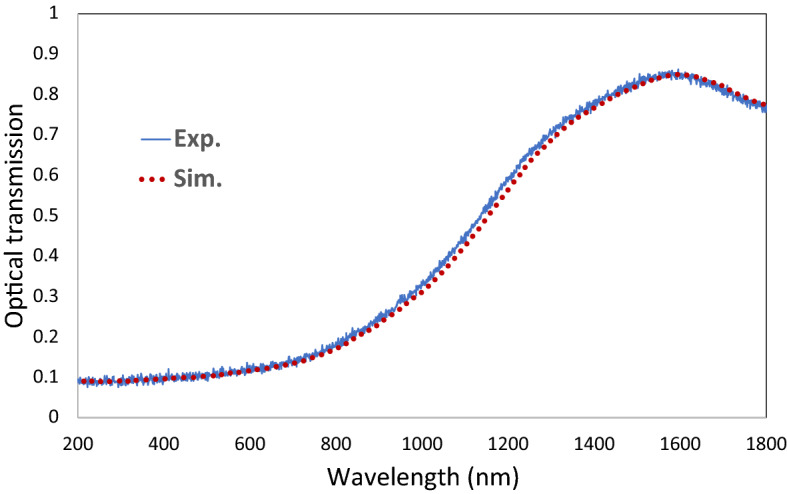
The UV–Vis-NIR numerically simulated normalized transmission spectrum using MEEP for BaTiO_3_, with the experimentally measured spectrum for validation. The dispersion and extinction were inputted using LD fitting coefficients.

Finally, the thin film of BaTiO_3_ prepared over a BK7 glass substrate is optically characterized using the Cary 5 UV–Vis spectrometer, see Fig. 14. Samples are measured against the BK7 substrate as a reference. In parallel, The FDTD was used to simulate a thin film of 178.45 μm thickness with two Gaussian beams centered at 550 nm, representing the TE and TM waves. As highlighted earlier, the material dispersion and extinction LD coefficients were captured from the DFT model. We utilize the same procedure as in^50^ for lithium titanate. The dispersion and extinction LD fitting coefficients were calculated to be: ${\upvarepsilon }_{\infty }$ = 1.595, ${\upsigma }_{1}$ = 1.5645 e + 41, ${\upomega }_{1}$ = 0.1254, ${\Gamma }_{1}$ = 0.05656, ${\upsigma }_{2}$ = 7.5565. ${\upomega }_{2}$ = 0.55128, ${\Gamma }_{2}$ = 1.9788, ${\upsigma }_{3}$ = 0.78155, ${\upomega }_{3}$ = 2.564578, ${\Gamma }_{3}$ = 2.2251, ${\upsigma }_{4}$ = 0.17521, ${\upomega }_{4}$ = 1.78215, ${\Gamma }_{4}$ = 0.95448 ${\upsigma }_{5}$ = 0.05551, ${\upomega }_{5}$ = 0.74158, and ${\Gamma }_{5}$ = 0.01452.

For experimental validation, the T-λ FDTD simulated spectrum is demonstrated against the UV–Vis-NIR spectrometer measurement for our fabricated thin film. For accurate matching, the thin film scattering prefactor was considered the main free-fitting parameter with the given material thickness and refractive index (as a function of the permittivity previously simulated using DFT). A thin film of 178.45 μm thickness was reached. The comparison between the simulation data and the experimental measurements in Fig. 14 indicates an acceptable argument with an average root-mean-square error of 1.44%.

## Conclusion

In conclusion, this paper introduces an experimentally validated FDTD numerical model to describe the optical properties of BaTiO_3_ as a potential layer in optoelectronic devices. Firstly, the DFT VASP model was used to estimate the energy band gap, nearly 3.21 eV, with DRS optical measurement agreement. The permittivity's real and imaginary parts of the permittivity were calculated, showing a refractive index of 1.595. Consequently, LD fitting parameters were used to input the complex permittivity of the BaTiO_3_ into the MEEP model. Less than 1.44% error was observed while the simulated MEEP spectrum was compared with the corresponding experimental spectrum. Experimentally, the manuscript provided a novel recipe to prepare and deposit a thin film of BaTiO_3_ with all essential morphological, physiochemical, and optical characteristics. A further investigation of other electrical properties, specifically the magnetic and the hall effect properties, can be part of future work.

## Data Availability

The data that support the findings of this study are available as follows: https://www.mathworks.com/matlabcentral/fileexchange/76474-dssc-optical-modelling. Any other data supporting this study's findings are available from the corresponding author upon reasonable request.
